# Duality of roles and moral injury in defence force nurses

**DOI:** 10.1177/09697330251366598

**Published:** 2025-09-04

**Authors:** Kristina Griffin, Therese Taylor

**Affiliations:** 110481 Charles Sturt University Faculty of Science and Health; 268549 Charles Sturt University Faculty of Arts and Education

**Keywords:** moral injury, ethical conflicts, defence force, role duality, deployment, Veteran suicide

## Abstract

**Background:**

Military medics, who are both professional soldiers and qualified nurses, can face situations where their training and moral ethos conflict in the performance of duty. Their role has intrinsic duality. They are both a soldier, thus a member of a military organisation as well as well as a healer, a nurse with a corresponding duty of care. Both roles have ethical, legal and professional responsibilities, codes of conduct and moral codes. Both also are roles which have strong cultural images and distinct expectations from individuals and those around them. This can lead to moral dilemmas, moral injury and long-term psychological illness. In the 2024 Royal Commission into Defence and Veteran Suicide in Australia, moral injury was cited as a relatively new, but not widely acknowledged, risk factor that may lead to suicide. In the context of defence, moral injury can be described as being experienced by a person who participates in, or witnesses, actions that go against their moral code or moral beliefs.

**Research design:**

The research presented is a component of a larger study into the role duality of the Australian Army medic in warzones. It focuses on interviews from twelve currently serving army medics who have deployed overseas in recent conflicts with the research question: How does the intrinsic duality of the role of the medic impact the individual performing this specialised role.

**Ethical considerations:**

This research has ethics approval from both the Australian Defence Human Research Ethics Committee #778–15 and the Human Research Ethics Committee #2015/024. Participants have given formal consent for their reflections to be used in publications.

**Conclusion:**

For this research the psychological impact of these ethical conflicts on Australian Army Medics who have served in this dual role has been investigated, with specific focus on moral injury.

## Introduction

The contemporary Australian Army medic holds dual qualifications. They are trained soldiers and are also registered with the Australian Health Practitioner Regulation Agency (AHPRA) as enrolled nurses. This results in legal and professional responsibilities and overarching codes of ethical conduct that create dual responsibilities. This can result in professional and ethical conflicts for the individual and moral dilemmas which may create long lasting psychological distress. In this article, the duality of soldier and healer is analysed through the testimonies of serving Australian Army medics. They have been interviewed for research into the history of nursing in the Australian Defence environment. Their testimonies are a primary source. Analysis of their recollections offer insight into the subjective experiences of ethical and moral challenges, both in the immediacy of events and on reflection. This article will show that the emotional and psychological impact of a duality of roles is profound. The memories are contextualised with writings from other secondary sources to uncover both short- and long-term themes such as regret, moral injury and recovery. The experiences and self-definition of soldier medics are foregrounded, and the consequences of the unique nature of their service is explored. This article considers not what medic services meant to the war, but what war meant to the medic.

Twelve currently serving Australian Army medics who had served in Timor-Leste, Papua New Guinea, Banda Aceh, Iraq and Afghanistan were interviewed and tell their stories. They were prompted by set questions but were free to choose their own emphasis and speak freely. They discuss the role duality and ethical challenges faced serving as a both healer and warrior in conflict zones, and its personal impact. The unique responsibilities of the army medic gives them professional identities across several fields. They are nurses and soldiers. They have also have experiences in the larger categories of warrior and healer. All of these terms are used in this article, and we hope to show that the medics are liminal figures who enact contradictory roles.

## Research method and sources

The army medics interviewed are deidentified for the purpose of this article and are referred to by initials. All the primary source material for this article – the recorded and transcribed interviews – are held by the researcher. The interviews were conducted through a process reviewed by professional ethics committees of both University and the Defence Forces. The names of some of the informants cannot be disclosed.

The interviews, once completed and transcribed, were found to be a rich source of information about the immediate experience of military service by medics. The numbers interviewed were small and that was in keeping with the aim to conduct in-depth conversations. All the themes in the interviews have been contextualised through reference to the academic literature on military history, as well sources discussing the recent fields of conflict where Australian Defence forces have served.

The methodology of the research was drawn from the philosophy of William Dilthey, who recommended that all historians enter into the life world of those whom they write about. He steers writers of history ‘in the direction of understanding and away from the notion of knowing’.^1^

This is a hermeneutic approach which gives authority to those who have lived through historical moments. Throughout this article, precision and analysis is accompanied by respect for subjectivity and individual perspectives.

## Discussion

War has been described as the ‘antithesis of health on all levels; spiritual, physical, psychological and economical’.^2^ It poses unique challenges for medics on overseas deployments where they are sometimes described as being a ‘warrior nurse’.^2^ Literature has revealed that this constant role duality, ethical dilemmas and exposure to death, creates an environment where these specialised soldiers are at higher risk of psychological injury than other service personnel.^3^ Healthcare professionals, military and civilian, have ethical, professional and legal accountability, owing a duty of care to their patients regardless of location.^4^ The army medic, who is recognised as a civilian nurse, is therefore guided by nursing codes of ethics^5^ and professional conduct^6^ which provides a framework for patient care and professional decision making.^7^ These may rival military objectives and orders of battle, leading to a disaccord for the person involved and result in a moral dilemma.

A quote from Gross gives insight into the dilemma that may be experienced by the army medic when performing his duties in line with military ethos ‘[O]n the battlefield, military healthcare is an adjunct of war, it does not speak to saving the lives of soldiers as an end in itself, but of salvaging their lives so they can fight’.^8^ This indicates that care provided may conflict with primary nursing ethos, providing care not for the best interest of the patient but rather in the best interest of the battle. It could even contravene the Professional Code of Conduct for Nurses that mandates patient care be performed in an ethical way, adhering to healthcare standards, while stating that breeching these could be considered professional misconduct. This adds to the moral dilemmas faced by the medic in provision of care.

In 2024 a report on the findings of the Royal Commission into Defence and Veteran Suicide in Australia was released to the public. This report is the culmination of 3 years of enquiry into the suicide crisis in Australia’s Defence and veteran population where the number of deaths is unacceptably high.^9^ The comprehensive report analyses cultural and systemic issues within the Australian military. The Report offers recommendations for reform to address the frightening trend towards ever higher rates of suicide. The Report offers suggestions for support of Australian Defence force members, both current and retired.

As a document which has recently become available, the Report of the Royal Commission is not yet present in academic texts. The first published study of its findings have been given by Effie Karageorgos, PhD, a social historian at the University of Newcastle, Australia. Her chapter ‘Locating the “moral genealogy” of War Trauma in the Australian Royal Commission into Defence and Veteran Suicide’ compares the current emphasis on mental health to previous approaches which blamed the problems of ex-service members on individual factors. Dr Karageorgos’ study is informative of how military health care services negotiate their stated mission amid ideological and nationalistic imperatives.^10^

In the report ‘moral injury’ was cited as a relatively new, but not widely acknowledged, risk factor in causing psychological injury that may lead to suicide. In the context of defence, moral injury can be described is as being experienced by a person who participates in, or witnesses, actions that go against their moral code or moral beliefs.^11^ Exposure to traumatising events on operational deployments, both war-like and non-combat, leads to psychological distress, such as Post Traumatic Distress Syndrome (PTSD).^9^ Both these are applicable in the case of the military medic; however, they are different. PTSD usually results from a traumatic event that predominantly involves a threat to life or safety, triggering strong fear-based emotions. Moral distress need not be personally threatening, but rather trigger emotions of shame and guilt^12^ and is not as well understood or documented as PTSD. It has been described as a ‘wounding of the soul, of doing something or witnessing something really wrong’.^12^

This is a significant factor for military medics who have two sets of ethical codes, often conflicting. The following of one whilst ignoring the other leads to a violation of these codes.^11^ Violation of moral codes can lead to moral distress and moral injury which can manifest in psychological trauma, anxiety, depression, self-harm and other symptoms.^13^ It can be exacerbated for the medic if coupled with feelings of blame and guilt if unable to perform their primary role of saving lives.^14^

A 2022 review on moral injury by the Department of Veterans affairs examined adverse outcomes for members of the military that included emotional, psychological, and interpersonal harms that could become debilitating. Early identification and assessment is vital to minimise impact.^15^ A significant recommendation in the Royal Commission report is to: ‘Prevent, minimise and treat moral injury’ which includes the need for further research into gaining a better understanding of the triggers and impact of moral injury in the Australian military population.^9^ This has significant implications for the research presented here as moral distress is linked to violation of a person’s moral or ethical code which is of particular significance to military nurses.^9^ The identification of triggers could lead to improved training and support, as recognition and preparation for ethical challenges leads to improved outcomes.^16^

When examining the role conflict faced by military medics, it is important to understand that conflict can arise when there is a discrepancy between a person’s moral beliefs and what they experience, causing distress. These discrepancies may arise from one’s own actions, or witnessing the actions of others, causing dissonance.^17^ For the military medic who holds two sets of moral imperatives, this conflict can be extreme. If they must make difficult professional decisions that transgress their valued beliefs,^16^ or witness actions such as violence or other inhumane acts by others who serve alongside them, it can lead to morally injurious events and associated trauma.^9^ Considering the severe negative consequences associated with moral injury, recognition of factors leading to these dilemmas and potential interventions is vital.^18^

There is limited data on the severity, prevalence and impact of moral injury amongst Australian Defence members. However, in 2021 a review of the impact amongst United States (US) military personnel found that 90% of US veterans reported at least one symptom associated with moral injury.^19^ As it is widely accepted that the early identification of mental health issues leads to more positive outcomes, identification of moral injury before it becomes extreme is important.^20^ To support this, research such as presented in this article into causes of moral distress and moral injury are vital in raising awareness and developing support and treatment options as there has been found to be a strong association between moral injury, depression and suicide in military and veteran populations.^15^ The specialised focus on army medics, in this article, links to the larger theme of veterans experiences, and contributes to an understanding of both.

A significant presence of non-uniformed enemies or potentially hostile civilians in zones of war increase the risk of moral injury.^21^ Nurses have professional code of ethics that outline standards and values to support decision making in the care of patients, and the upholding of human rights.^5^ These value statements recognise the relationship between health and human rights,^22^ dictating that the nurse will care for all equally. However, this becomes impossible when those requiring care are potential enemies in the combat environment. In these situations, military medics are unable to make the patient their first concern, as they are also a soldier, and may thus breach their other responsibilities. Military nurses duty of care in conflict situations may be constrained by military orders or risks to their own comrades.^23^ If these orders conflict with their duty to assist casualties they will be faced with an dilemma as to what action to take,^23^ and may also face military punishment if they fail to obey orders,^24^ causing greater conflict and stress.

Thus, the military medic is bound by two sets of doctrine within their dual roles of soldier and nurse. The resultant ethical dilemmas may be difficult to reconcile if in conflict with each other. In a civilian medical emergency, a nurse has a clear priority of patient care. However, in a battlefield situation the military nurse is part of the organisation involved in the fighting, causing challenges to the delivery of care^25^ and a conflict in governing roles. Military orders can impede the medic from making decisions related to patient care. Tripodi believes these dilemmas are overcome by the medics military training,^26^ however the recommended actions may breach nursing principles, challenge ethical convictions and possibly arouse feelings of guilt or shame.^27^ They can be acting legally and ethically according to one set of guiding principles, whilst compromising another, as responsibilities diverge.^28^

Treatment of casualties may become a source of ethical conflict when there are wounded from both allied and enemy forces and the need for medical attention exceeds resource supply. Conflict can arise as to who is treated first with medical neutrality in conflict with military strategies^29^ as military medical personnel have obligations to both patients and the defence force.^30^ These dual-loyalty obligations of the medic who both preserve life as a health professional, but supports the infliction of harm as a soldier,^31^ are often irreconcilable.^32^

An examination by Frisina on military medical ethics has discussed three fundamental ways that values may be developed intrinsically by military medical personnel, causing ethical dilemmas if principles clash. These are: 1). An individual’s personal ethics, 2). Professional ethics and values from the medical training received and professional body, 3). The military system and its values.^33^ As discussed previously participating in or failing to prevent acts that transgress moral beliefs can lead to moral injury and resultant psychological trauma.^11^

The Australian Military Medics that have shared their stories for my research echo these reflections, especially in relation to recent conflicts in the Middle Eastern Area of Operation (MEAO) of Iraq and Afghanistan. Here feelings of self-doubt arose in conflicts where the enemy is at times unknown, and the combat convoluted. These wars have been controversial and met with protest, debate and diverse public and media opinion. Beliefs about Australia’s involvement in these hostilities range from support for humanitarian efforts to opposition of participation in what was seen as a politically motivated combat.^34^ This public negativity adds to the emotional stress felt by the combat medics.^35^

## Themes

### Identity dissonance

A comment by Tom Frame, in his writings on moral injury, reflects the ambiguity and confusion felt by those involved in the MEAO: ‘we are fighting on behalf of allies that seem as bad as the enemy, why is it my responsibility to fight in foreign lands to keep peace between people disinterested in peace’.^27^ A specialist in the ethics of armed combat, Deane-Peter Baker, comments that these recent conflicts where the enemy is unrecognisable, causes moral dilemmas for those fighting.^27^ T (interview, 2017), supports these comments and his own inner conflict:

*you don’t exactly know who the enemy is unless they are standing right in front of you trying to blow you up or shooting at you (…) so it is not a real clear cut line as to who we are fighting … friends who’ve been over previous to myself, that is what they said was an issue*.

JP (interview, 2016), commented that that this created a stressful and unpredictable environment when there was no real way of identifying the enemy ‘*not a clue because there is no uniform … you literally have no idea, so no real way to get over it, some people see everyone as the enemy’*. This was exacerbated when soldiers from the Afghan National Army (ANA) attacked members of coalition forces in 2011, intensifying the fear of the unknown and the moral quandary; leading to moral injury and psychological distress.^36^

Self-identity for the military medic who has significant role duality between soldier and nurse may be central to the moral conflict felt by the individual. Some interviewees saw themselves as primarily a soldier, with an additional role of providing life-saving interventions when their team requires it, others see the provision of healthcare as paramount and their role as a soldier to protect themselves and the wounded.

T (interview, 2017) describes himself as a soldier, capable of defending himself and those around him stating ‘I* think that if you are trained in all weapons that you should be able to use all weapons to the best of their capability. If it means that a life or death requires you to protect yourself and all those under your direct care, then you do it’*. JP (interview, 2016), had a similar view describing himself as a ‘*soldier* *first*’, reflecting ‘… *that does not mean I am a war fighter, kicker, but I am definitely a soldier. With that my speciality role is to be a clinician*’. GM (interview, 2016), had a similar perspective relating his views as well as his perception of a role change for the army medic in recent times:

*my attitude is that you are also a soldier first and foremost so, despite the fact that your role is providing medical care, you’re also the person on the front line firing as required, if required. That is part of the job. If someone is shooting at you then you should shoot back at them (…) This was a very different role to what we used to do when we would say [pause], no, we are a medic, we don’t shoot. [pause] This is not true anymore.*

These reflections indicate a military focused self-identity that may assist with compartmentalising their conflicting responsibilities and moral codes, or they could be a way of justifying actions to resolve inner conflicts that arise from differing ethical perspectives and experiences of their role duality. MC (interview, 2017) describes this role ambiguity and need for role transition when he states his beliefs:

*It is not my role to go looking for a fight; however as a soldier my job is to be prepared to fight until I received casualties, then I become a medic (…) you go overseas with the rifle and real bullets, you should be prepared to use them. [pause] Again, if you're a medic on a battlefield in a warzone it’s plain reality.*

DP (interview, 2017), conversely, after years as both a military medic and civilian registered nurse, aligns himself more as a nurse than a soldier stating: ‘*I didn’t feel I wanted to shoot a person’*. Despite this he understood the importance of carrying a weapon to protect himself and his patients, qualifying *‘I have seen the results of not being able to defend yourself … in the conflict in Rwanda, guys got killed because they had no protection’*. He is referring to the violent massacre of refugees in 1995 in Kibeho, Rwanda where Australian troops were sent as part of a humanitarian aid contingent.^37^ This may have a significant impact on DP as his morals as a healer have to be pushed aside as he engages in combat as a soldier, despite his reluctance.

### Post-deployment dislocation

All these reflections demonstrate role duality, requiring the individual to cross boundaries and responsibilities. This necessitates compartmentalising of identities and suppression of solid belief systems, requiring each separate role to be personally and individually negotiated.^2^ The impact this has on the interviewees transition home is discussed later but must also create dilemmas that possibly have long-term psychological effects from what is now described as moral injury.

Deployments potentially cause stressors and traumatic experiences for all military personnel.^38^ The medics in this study were asked how deployment overseas as part of a fighting force affected them, in both the short and long term. AW (interview, 2017), commented the significant impact of deployment on him personally: *‘absolutely [pause] I am still dealing with it … I went over there pretty inflated, thinking I could do anything, but I feel deeply responsible for a death over there. I missed a patient that was decompensating. [pause] That hurt me and it still hurts me’*. This indicates the deep psychological injury that his deployment as a medic has caused.

### Moral dilemmas

T (interview, 2017), felt conflict on return home, related to his role overseas that sees a morality conflict between his dual roles of soldier and healer:

*I suppose a lot of it comes down to what we are there to do in our job. It is effectively to find and get rid of the* *so-* *called* *enemy as such and if they are of a certain religion or certain race, certain ethnic group then yes I think there is always going to be hate. That hate sometimes comes home with* *you.*

This testimony acknowledges the hostility which thrives behind the facade of warfare for noble causes, such as peace keeping or eradicating terrorism. The medic is not able to leave ethnic and sectarian animosities behind when he leaves the conflict zone. But an attitude of hate toward other races or religions transgresses the nursing Code of Conduct in Australia. It is notable that other medics had an affectionate view of the Afghan people whom they encountered, but their empathy with the civilian population only increased their stress, because they could do little to assist them.

T (interview, 2017) reflected that negative memories were exacerbated by feelings of futility as he was unable to protect his own team as a soldier or keep them alive as a medic. He described the worse part of his deployments as:

*I’d guess the worst thing is doing what you are trained to do but to nil effect. So I guess having people die, feeling pissed off and angry that you can’t even look after your own, effectively that we couldn’t do what we said we could do [pause]: keep them alive or look after them after that*.

MC (interview, 2017) became very emotional when expressing similar feelings of moral injury when he could not perform his primary role as a medic: saving the lives of those in his care. He recounts an incident in Afghanistan where his dual roles of soldier and healer are personified as he unsuccessfully attempted to save a member of his company whilst at the same time in combat as a soldier, fighting the enemy. They were under attack and all lives were in danger. This incident had a profound effect on MC who, when asked if he wanted to come home after his deployment stated ‘*no, I wanted to stay as I felt I had a job still to do’*. This statement shows the unresolved feelings of a soldier who cannot easily leave the conflict zone, because of a sense of incompletion.

Indistinct military actions that encompass combat duties, peacekeeping and also humanitarian aid are inherently challenging. Furthermore, involvement in conflicts in the MEAO where the enemy combatants resemble civilians, causes complexity and confusion. Deployments in these environments increase the psychological impact on military healthcare workers when caring lines are blurred with the need for safety.^39^ This confusion creates a risk of moral injury when morality and conviction are challenged and ethical lines are blurred.^40^

### Returning home

Returning home is the moment when thoughts and feelings become memories. As such, these mental images of war can come to dominate consciousness. Transitioning home from a conflict environment that has caused psychological stress or injury is challenging. This process of return is supported by military procedures, but are they adequate? T (interview, 2017) does not believe so ‘*I personally think, know, there is not enough time to get prepared to come home’*. Continuing, he discussed the services available:

*A lot of people don’t want to speak to the Defence or the army psychologists, and I know a lot of people including myself have wanted to go and speak with someone on the outside of Defence (…) it is a comfort thing, not speaking to someone in uniform. I don’t think they [civilian services] understand completely the ins and outs, but they have a different look on it which helps.*

According to MC (interview, 2017), the loss of the soldier in his care he did not find the psychological support offered of benefit. On return home he struggled. He reflected that the incident impacted him. He was unable to process what had happened and he switched off. He commented that he had relationship issues with his partner ‘*she thinks I’m emotionless’*. Interestingly, he displayed significant emotion during his interview, indicating the inner psychological struggle and trauma that he was attempting to deal with. He also spoke of himself in the third person in his reflections, possibly indicating emotional compartmentalising, or disassociation, to allow him to cope.

Stephen Garton, in his writings on the effects of war on Australian veterans, spoke of emotional distancing as an attempt to forget, in order to be able to move on, rather than to confront the pain and horror.^41^ JP (interview, 2016), spoke of this compartmentalisation in order to cope with what was happening whilst on deployment commenting: ‘*Exposure is going to change you, conflict is going to change you (…) you get very good at compartmentalising things to cope, far too good, to the point it became a problem, big, big problem when I came back’*. This shutting off of emotions to allow him to do his job became an issue and made his transition home complex. He commented that he became *‘maladaptive’* and ‘*lacking in emotion’*.

If support or understanding on return is lacking, psychological issues are exacerbated.^27^ JP (interview, 2016), reflected that whist in the combat environment he was comfortable with the decisions he made, but on return his actions were questioned by others or attracted negative media attention, causing him to over scrutinise his own actions. He stated he started to overthink his actions:

*Why did I do that? Well I thought I was doing a good thing. [pause] But you get all this hatred and then you start to over-think. That tends to put you into a bit of a spiral, basically, as you start to over-analyse and question what you’ve done and over-think. That leads to depression and all sorts of maladaptive disorders basically, and then poor choices and that compounds it.*

This reflection indicates clearly the psychological torment felt by these nurse warriors. The sense of stigmatisation is evident, although it cannot be quantified, or even measured consistently in one individual. However, it is observed in all discourse around the return of soldiers from recent controversial conflicts in the Middle East.

The psychological stress is exacerbated by the medics’ feeling of not being needed on return home. After a period of deployment where they are responsible for the lives of their company, they return to a life and family that have moved on, causing feelings of inadequacy and uselessness. JP (interview, 2016) expressed:

*I came back as this robotic person that wanted to be an asset in the house that realistically I wasn’t needed any more (…) you are not required anymore because they have done everything without you, so [pause] there was that messy period of readjustment.*

Feelings of worthlessness and inability to understand that people can only do their best leads to an inability to cope, according to AN (interview, 2016), *‘they feel worthless, and it’s that feeling of worthlessness that leads to them ending up developing PTSD or some sort of depressive disorder’*.

Disconnection from the horrors of war, in order to cope psychologically, is vital but difficult. An Australian Army Medic, Terry Ledgard, describes the process as ‘disengaging from the confronting nature of war is an acquired skill that I still need to develop’, indicating his inability to leave combat behind.^42^ Feelings of a job left undone, impact of moral challenges, and the loss of lives, coupled with an inability to unwind or disclose their thoughts and feelings, are consistently found in all sources. When asked if she was prepared to return home SK (interview, 2017) commented: *‘Emotionally at the time I would have said yes, but now I look back at it I don’t know how one could prepare people emotionally to reintegrate back into normal civilian life’*. These underlying stressors can cause psychological distress that may be transient, or may lead to the development of permanent mental health issues.

This is a theme common to returned soldiers throughout history. Garton discusses the process of repatriation for World War Two (WWII) soldiers, that had significant gaps, as institutions took care of essentials but failed to address individual needs. These men struggled to return to a normal life with their psychological demons exacerbating their problems.^41^ The moral injury felt by our latest returned veterans reportedly remains unsupported. It is recognised that military service in modern conflicts requires soldiers to act in multiple roles including humanitarian aid, peacekeeping operations and combat. Compounding this is the added burden of multiple deployments, loss of unit members and the witnessing of immoral acts that challenge an individual’s values and cause mental health issues to arise.^15^ To avoid the severe mental health outcomes that result, and promote recovery, appropriate holistic psychosocial care is needed.^43^

There are barriers, perceived and real, that can negatively impact veterans seeking the support they require. There is an identified stigma within the military in seeking psychological support, as it can be viewed as a form of weakness.^15^ Negative attitudes toward help seeking contribute toward high rates of psychological injury and poor outcomes. This coupled with feelings of decreased self-worth, weakness and shame at not being a fully functional team member and fear of career limitation adds to psychological injury.^14^

Moral injury can create self-stigmatising beliefs that are linked to increased suicidal behaviours and also to negative coping strategies used to numb feelings, such as drug and alcohol abuse, disconnection from personal and professional relationships and support services, and self-isolation; with suicide being the ‘ultimate disconnect’.^9^ Risk of exposure to traumatic events, that is inherent with military service, can be a predictor of psychological distress. Isolation from family and support networks are a constant factor in military service. However, it is the support offered post event and upon return home that affects long-term impact.^12^ Removing cultural and structural barriers to seeking help and reducing stigma is vital, as is further research into better understanding and the development of ways to minimise the effect of moral injury in the Australian military population. These are all recommendations of the Australian Government Royal Commission report into Defence and Veteran Suicide.^9^

For the medics who have served in recent conflicts such as Iraq and Afghanistan, who are my research participants, the rules that govern military operations become ambiguous in the field. Lines become blurred, causing ethical dilemmas and moral injury when soldiers feel they have made an incorrect decision.^44^ This is exacerbated by the sense of scrutiny brought about by headcam footage in current conflict zones. A soldier’s actions are recorded and can be brought forward in war crime investigations, as has recently occurred in Australia, adding to the psychological stress of deployment.^45^ Observation by others, as well as the interior dialogue of self reproach, contribute to a sense of stigmatisation.

## Conclusion

Moral distress and resultant moral injury from violation of moral codes can lead to both short and long-term psychological trauma. This manifests as anxiety, depression, self-harm and suicidal thoughts and intent.^21^ The risk of such harmful outcomes is increased and exacerbated through the role duality of the Australian Army medic. Moral injury is an unseen wound,^27^ a scar on the soul – not as well documented or understood as PTSD but deeply connected to suicide and suicidality of Australian soldiers.^9^ The effects are more pronounced for the soldier healer, the medic, as they struggle with decisions made or trauma witnessed that transgress their nursing ethics. It therefore requires greater research. This would lead to strategies of prevention and supportive treatment; not only for the military and veteran populations worldwide, but for all at risk of developing moral trauma and the associated debilitating effects.
